# Men’s Health in Northern British Columbia: Analysis and Reporting of Early Intervention Screening Program Data Related to Cardiovascular Health

**DOI:** 10.5539/gjhs.v4n4p90

**Published:** 2012-06-07

**Authors:** Mamdouh M. Shubair, Anthony L. Gagne

**Affiliations:** 1 School of Health Sciences, University of Northern British Columbia (UNBC), Prince George, BC, Canada

**Keywords:** men’s health, type 2 diabetes, cardiovascular disease, northern British Columbia, risk factor survey, point of care testing

## Abstract

Type 2 diabetes (T2D) is a well-established risk factor for cardiovascular disease (CVD). Higher rates of T2D are attributable to unhealthy lifestyle factors and a number of clinical and metabolic risk factors. There is paucity of research which investigated the association of lifestyle risk factors and metabolic markers amongst adult men in northern British Columbia (BC). Using a face-to-face screening questionnaire, we assessed the relationships between age, body mass index (BMI), and a number of CVD risk factors in a convenience sample of 123 eligible men recruited from communities across northern BC in February of 2011. In regards to the metabolic risk factors measured through screening blood tests (lipid profiles; blood glucose) responses to the questionnaire were dichotomized into high and low risk categories. These dichotomized variables were subsequently used to determine if significant associations existed with each of the age category variable and a standard BMI categorical variable. There were significant linear relationships between the categorical BMI variable and a number of metabolic risk factors, as well as smoking history. Older age (40^+^ years) was associated with higher BMI status (overweight/obese). Our findings provide compelling evidence that northern BC men possess a number of clinical, metabolic, and lifestyle risk factors associated with high CV risk. Future studies should examine other sociodemographic variables including occupation status, education attainment, and ethnicity, and other psychosocial determinants which include knowledge, attitudes, and perceptions (KAP) related to T2D and CV risk profile in adult men working and living in northern BC, Canada.

## 1. Introduction

The emerging global epidemic of obesity, combined with dietary factors, sedentary lifestyle, and genetic factors, has led to many societal and physical consequences; possibly the most detrimental of these physical consequences is increased incidence of cardiovascular disease (CVD) (Grundy, 2004; Hu et al., 2004). In 2007, 4.8% of Canadians over the age of 12 years reported having a diagnosis of heart disease, with men being more likely than women to have been diagnosed with this disease (Heart and Stroke Foundation of BC & Yukon, 2011). Despite differences in rates of CVD among both sexes, there is paucity of research examining risk factors of CVD in men. Furthermore, most of the published studies have been conducted in the United States and Europe, leading to concerns of external validity and applicability to men in northern British Columbia (northern BC).

As aforementioned, obesity is considered a well-established risk factor, as it contributes to the development of CVD by influencing several major and emerging cardiometabolic and non-metabolic risk factors (Bays et al., 2007; Grundy, 2004). These cardiometabolic and non-metabolic risk factors include hypertension, dyslipidemia, and insulin resistance, which are collectively known as the Metabolic Syndrome (MetS) (Lakka et al., 2002).

In addition to the biological risk factors which make up the MetS, there are several lifestyle and other risk factors that have been shown to contribute to the development of CVD. For example, some studies have indicated that smoking history may influence the development of symptoms associated with the MetS and is therefore considered an underlying risk factor (Hu et al., 2004; McNeill et al., 2005). Age has been shown to be associated with an increase in prevalence of the MetS, with the highest rates seen in those above the age of 60 (Hu et al., 2004; Regitz-Zagrosek et al., 2006). However, the prevalence of the MetS is also high among those less than 60 years of age. For example, a study found an upward trend in rates of the MetS in obese adolescents, with males showing a much greater risk than females (Duncan, Li, & Zhou, 2004).

Several studies have been carried examining the relationship between prevalence of the MetS and CVD in a number of large populations. In one study, Lakka and colleagues (2002) determined the prevalence of the MetS in a cohort of middle aged Finnish men and tracked them for 14 years to determine mortality from CVD, coronary heart disease (CHD), and all-cause mortality. After follow-up, it was found that participants who were diagnosed as having the MetS at baseline were up to three times more likely to have died from CVD over the course of the study than contemporaries who did not have the MetS at baseline (Lakka et al., 2002). Subjects diagnosed with the MetS were also found to have a significantly increased risk of CHD mortality and all-cause mortality compared to those without the MetS, even after potential confounding variables such as age, smoking history, and family history of CHD were controlled for (Lakka et al., 2002). This finding was also supported by a large-scale meta-analysis of studies conducted on European cohorts (Hu et al., 2004).

Another study examined risk of developing CVD associated with the MetS in a community-based population sample in the United States (McNeill et al., 2005). The prevalence of the MetS was assessed among a large cohort followed for an average of 11 years, with incident CHD and stroke events assessed. The results of this study indicated a positive relationship between the MetS and incident CHD cases and stroke events in male study participants. This association remained significant even after controlling for potential confounding variables. Furthermore, McNeill et al. (2005) were able to identify a significant positive association between risk of developing CHD and the number of risk factors associated with the MetS present at baseline.

To our knowledge, there have been no previous studies which assessed risk factors for CVD and associated sociodemographic correlates among men in northern BC. The objectives of the current study were: a) to collect information through a self-report face-to-face screening questionnaire on known behavioural (lifestyle) risk factors of CVD amongst men living in northern BC, as well as measure biological (cardiometabolic) risk factors for CVD (blood pressure, cholesterol/lipid profile, blood glucose); and b) assess relationships or associations among age, body mass index (BMI) and other risk factors for CVD using contingency table analysis (Chi-squared tests).

## 2. Methods

### 2.1 Measures and Procedures

Information on risk factors associated with the MetS and CVD were collected using the *CHECK-MATE Early Intervention Screening Questionnaire* (Appendix A). This community-based screening assessment tool has been implemented successfully in rural Australia (Verrinder & Denner, 2000; Shephard et al., 2005). This brief 10-item questionnaire instrument was designed to collect data on certain CVD risk factors. These include: personal history of heart disease; family history of heart disease; smoking status; blood pressure; cholesterol level; diabetes and blood glucose levels; weight/BMI; alcohol consumption; physical activity habits; and stress levels. The questionnaire is designed to yield a risk score ranging from 0-10. For the purposes of the current study, this screening assessment tool has been adopted by our strategic community partner; namely, Northern Health Authority (NHA). The researchers (authors) of this study (Shubair; Gagne) were invited to be involved in this research project after the data collection phase, as expert consultants on the data analysis and reporting. Quantitative data related to blood pressure, cholesterol levels, and blood glucose was collected by trained public health nurses from Northern Health. Such measures were collected using point-of-care testing protocols as opposed to standard laboratory procedures. Weight and height were self-reported by study participants. Information solicited in relation to personal and family history of CVD and associated risk factors (such as family history of diabetes; family history of heart attack) were self-reported. Behavioural lifestyle factors that were also self-reported included smoking, alcohol consumption, physical activity, and stress levels.

### 2.2 Participants

Study participants were recruited through convenience sampling between February 7^th^ and 25^th^ 2011 in several communities across northern BC (Table 1). Trained interviewers attended a number of well-advertised community events including men-themed restaurant nights and sporting events, and recruited interested men who volunteered to complete the questionnaire (Appendix A). There was no specific exclusion criteria as all adult men (18 years of age or over) were eligible to participate in the study by completing the questionnaire after informed consent. A total of 161 individuals completed the questionnaire. There were 38 questionnaires determined to be ineligible at the time of data analysis because participants did not specify their gender (n=14), or selected their gender as female (n=24). For the purposes of statistical analysis in the current study, the final sample consisted of 123 subjects. The study protocol was approved by the Research Ethics Boards (REBs) of the University of Northern British Columbia (UNBC) and Northern Health Authority (NHA).

**Table 1:** Survey date and location

Survey Date	Survey Location (City/Venue)
February 7th 2011	Prince George/Train-the-Trainer Session (n=1)
February 10th 2011	Prince Rupert/Train-the-Trainer Session (n=9)
February 15th 2011	Prince George/Coast Inn Men’s night (n=25)
February 17th 2011	Prince George/Twisted Cork (n=13)
February 19th 2011	Prince George/CN Center (n=30)
February 21st 2011	Fort Nelson/Train-the-Trainer Session (n=9)
February 23rd 2011	Fort St. John/On the Rocks Men’s Night (n=7)
February 25th 2011	Prince George/Native Friendship Center (n=5)
Unknown	Unknown (n=24)

### 2.3 Data Analysis

All data were entered into SPSS 19.0 for Windows statistical package. The mean and standard deviation (mean ± SD) were determined for all continuous variables: blood pressure (systolic and diastolic); total cholesterol; high-density lipoprotein (HDL); low-density lipoprotein (LDL); HDL/LDL ratio; triglycerides level; random blood glucose levels; and BMI.

We conducted descriptive univariate analysis which included sample frequencies for all continuous and categorical variables in the survey data. Contingency table analysis (Chi-squared tests) were used to outline potential differences in the distribution of variables which included: personal and family history of heart disease; smoking history; use of medications for blood pressure, serum cholesterol, heart disease, and diabetes; personal and family history of diabetes; self-perceived weight; alcohol consumption; weekly physical activity; and stress. A probability value of *P* ≤ 0.05, with a two-tailed distribution, was considered indicative of statistical significance.

In order to carry out bivariate correlations, we dichotomized certain variables of interest based on probability of cardiovascular (CV) risk. This was carried out following the risk levels given in the questionnaire, which were in accordance with the WHO diagnostic criteria for the MetS. The following dichotomous variables were used for analysis: personal history of heart disease (0 = no personal history, 1 = having a personal history); family history of heart disease (0 = no family history, 1 = family member having heart disease); age (0 = participant < 40 years old, 1 = participant > 40 years old); smoking status (0 = never smoked, 1 = current or former smoker); blood pressure (0 = blood pressure < 140/90 mmHg, 1 = systolic blood pressure > 140 mmHg OR diastolic blood pressure > 90 mmHg); total cholesterol (0 = measured value < 5.5 mmol/L, 1 = measured value > 5.5 mmol/L); HDL Level (0 = measured value > 1.0 mmol/L, 1 = measured value < 1.0 mmol/L); HDL/LDL ratio (0 = measured value between 4.5 and 6.5, 1 = measured value < 4.5 or > 6.5); triglycerides (0 = measured value < 2.0 mmol/L, 1 = measured value > 2.0 mmol/L); personal history of diabetes (0 = no diagnosis of diabetes, 1 = having diabetes); family history of diabetes (0 = no family history of diabetes, 1 = family member having diabetes); BMI (0 = reported value < 27.0 kg/m^2^, 1 = reported value > 27.0 kg/m^2^); alcohol consumption (0 = low/moderate alcohol consumption, 1 = reported high consumption of alcohol); exercise (0 = any weekly physical activity reported, 1 = reported inactivity throughout a typical week); and stress (0 = reported low levels of stress, 1 = reported moderate or high stress levels). The HDL value of 1 was based on the community screening tool (Appendix A) developed by Denner (2009). Furthermore, the screening tool did not include a ‘non-drinkers’ category.

For the purposes of the contingency table analysis (Chi-squared tests) related to BMI, we derived a slightly modified WHO/NHLBI BMI categorical variable (BMI group) that combined class II and class III obese subjects because of small numbers as was done previously (Shubair et al., 2004). The Chi-squared tests were carried out to examine associations or correlations between each of: a) BMI group (standard BMI categories) and each of the dichotomous CV risk scores (see Methods); and b) Age group (as a categorical variable) and each of the dichotomous CV risk scores. A probability value of *P* ≤ 0.05, with a two-tailed distribution, was considered indicative of statistical significance.

## 3. Results

The analysis was based on 123 completed surveys. With the exception of BMI which was derived from the self-reported weight and height (kg/m^2^), Table 2 shows the mean ± SD of continuous variables which included risk factor data that were measured as part of the screening methods conducted at the various Men’s health screening events.

**Table 2:** Metabolic profile for study participants

Risk Factor (N)	Mean (SD)
Blood Pressure (Systolic) (n=106)	142.46 (17.61) mmHg
Blood Pressure (Diastolic) (n=106)	87.20 (11.71) mmHg
Cholesterol (n=110)	4.91 (1.55) mmol/L
HDL (n=109)	1.25 (0.51) mmol/L
LDL (n=87)	2.61 (1.05) mmol/L
HDL/LDL Ratio (n=99)	4.61 (2.07)
Triglycerides (n=109)	2.71 (1.50) mmol/L
Blood Glucose (n=112)	5.99 (2.02) mmol/L
BMI (n=57)	28.06 (6.29) kg/m^2^

**Figure 1:**
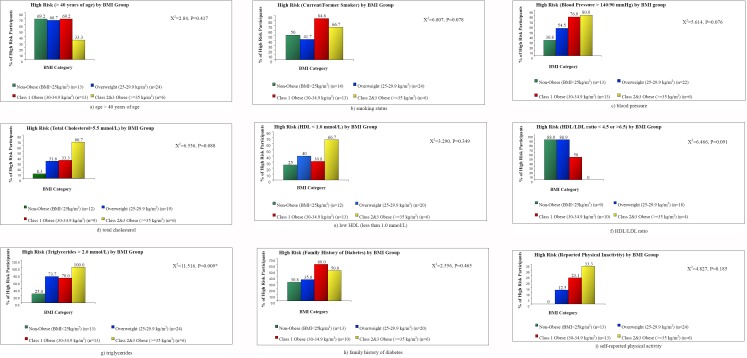
Percentage of participants with metabolic syndrome risk factors plotted against body mass index categories

**Figure 2:**
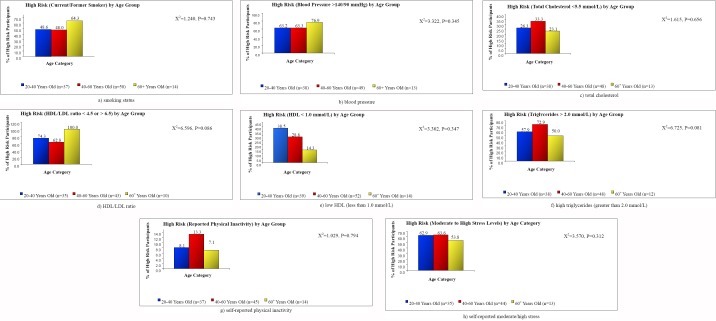
Percentage of participants with metabolic syndrome risk factors plotted against age categories

Cross tabulation was conducted between each of categorical BMI and age categorical variable and dichotomous risk scores (Figures 1 and 2). Chi-squared tests were calculated to examine the relationship between BMI group and each of dichotomous CV risk scores, and the relationship between age group and each of the dichotomous CV risk scores.

Results of the cross tabulation between BMI and the dichotomous risk scores were significantly different for triglyceride levels (*X*^2^=11.516, p=0.009). There was a significant linear trend between triglyceride levels across each of the BMI categories. As illustrated in Figure 1g, the proportions of participants with a significantly high triglyceride levels were 73.7%, 70%, and 100% for the overweight, class I obese, and class II/III obese respectively. When the relationship between BMI categories and each of smoking history (*X*^2^=6.807, p=0.078), blood pressure (*X*^2^=5.164, p=0.076) and total cholesterol (*X*^2^=6.536, p=0.088) was examined, we found a marginally significant linear relationship across the BMI categories (figures 1b-d). No significant associations were observed between age category and any of the dichotomous CV risk scores. However, a marginally significant association was seen between age group and triglyceride levels (*X*^2^=6.725, p=0.081). The highest triglyceride risk was seen in the 40-60 years old group, with 72.9% of participants in this group having a high risk score based on triglyceride levels (Figure 2f).

## 4. Discussion

The results of this study indicate that men who reside in northern BC possess several risk factors which have been associated with the development of CVD. Based on mean values, subjects in the current study are above the NHLBI set standards for blood pressure and triglycerides (Lakka et al., 2002) and are considered to be overweight based on the WHO/NHLBI criteria (Kuczmarski & Flegal, 2000). These risk factors are of great concern, as hypertension and dyslipidemia are both considered major risk factors of the MetS (Bays et al., 2007; Lakka et al., 2002). Many published reports have found significant associations between prevalence of the MetS and CVD morbidity and mortality (Bays et al., 2007; Hu et al., 2004; Lakka et al., 2002; McNeill et al., 2005).

With more advanced age, increased BMI was found to be associated with increased probability of having certain well-established risk factors of CVD and the MetS in the study population. This finding is not surprising, and is supported by previous studies. For example, Park et al. (2003) found a significant linear trend between BMI and prevalence of the MetS in both men and women, with obese men having the highest prevalence at nearly 60%. These authors also noted that the MetS was associated with more advanced age (Park et al., 2003). Another study in a cohort of First Nations participants found a significantly increased risk of CVD mortality amongst participants with higher BMI (Fang, Wylie-Rossett, Cohen, Kaplan, & Alderman, 2003).

In the current study, increased BMI was significantly or somewhat significantly associated with several metabolic and non-metabolic CVD risk factors. This finding has adverse health consequences or implications in terms of Men’s CV health in northern BC. It has been suggested that individuals who possess two or more risk factors of CVD are at higher risk of mortality from CVD compared with individuals who only possess one risk factor (Klein, Klein, & Lee, 2002). The finding that obesity is associated with multiple CV risk factors suggests that health promotion intervention strategies are warranted among men living and working in northern BC. We suggest that this finding has also policy implications in that upstream public health policy interventions which target obesity are needed given the multifactorial nature of obesity and its associated comorbid conditions such as type 2 diabetes (T2D), the MetS, and CVD.

There are a few limitations to the current study that need to be mentioned. While very few questionnaires were deemed ineligible, the majority of participants provided incomplete information. Responses for cardiometabolic risk factors (variables) varied from 112 subjects for blood sugar to 57 individuals for BMI. Another limitation pertains to the collection of the metabolic risk factors in that total cholesterol (TC), other lipid profiles, and blood glucose were not taken after a standard period of fasting (normally 12 hours fast). Caution should therefore be exercised while interpreting these data. A third issue relates to the inherent limitation of surveys in collecting self-reported data. In regards to BMI, it is possible that some men in the study sample may have misreported their weight and height. This is particularly relevant for older men, who have been shown to have a higher likelihood of misreporting their weight and height (Kuczmarski, Kuczmarski, & Najjar, 2001). As opposed to being self-reported, it would have been more valid and reliable to have weight and height data be actually measured. Finally, other than age there was no information collected on sociodemographic characteristics such as education, income, occupational (employment) history, and ethnic background given the diverse multi-ethnic nature of northern BC population (Hanlon & Halseth, 2005). It has been suggested that both employment characteristics (most notably job demands and job control) and socioeconomic status may play a role in risk of developing CVD in adult men (Lynch et al., 1997; Steenland, Johnson, & Nowlin, 1997).

Despite the small sample size and recruitment using convenience sampling (for feasibility reasons), we believe that the study findings are applicable to men living and working in northern BC. These findings, however, cannot be generalized to all men living in rural and remote northern regions of Canada. A future larger study with robust sample size should consider collecting such data in order to characterize the sociodemographic profile of men who are at-risk or high-risk of obesity and subsequent CVD.

## 5. Conclusions

Men living in northern BC possess a number of known metabolic and non-metabolic risk factors for obesity and CVD, many of which are also components of the MetS. Body weight status as measured by BMI category was shown to be significantly associated with several CV risk factors; most notably triglyceride levels, blood pressure, and total cholesterol (TC). Older age (40 years or over) was associated with higher BMI. While the findings from the current study are considered preliminary due to small sample size (n=123) and low response rate, the results provide compelling evidence to further examine other determinants of CVD including psychosocial factors such as knowledge, attitudes, and beliefs (KAB) of high-risk men population in northern BC as well as lifestyle factors in conjunction with fasting blood glucose and other metabolic markers for T2D and CVD. More specifically, the study of these risk factors among working men in the natural resources sector (forestry; logging; mining; oil and gas, and trucking industries) is warranted.

## Appendix A

The most important preventable heart disease risk factors identified by the National Heart Foundation are tobacco smoking, high blood pressure, raised blood cholesterol and blood sugar levels.

PERSONAL HISTORY

Do you have a history of heart disease? Yes◻ No◻

Are you on HRT therapy? Yes◻ No◻

**1 Age** Lessthan 20 ◻ **and Sex**

       20-40 ◻ Female ◻

       40-60 ◻ Male ◻

       60+ ◻

As you get older this risk of heart increases. Males are more *likely to develop heart disease earlier in life than females. Men 20 years^+^, women 40 years^+^ have an increased risk for heart disease*

**2 Family History**

Has any member of your family ever been diagnosed with heart disease? Yes ◻ No ◻

A family member had a heart attack or heart surgery

(i) over the age of 65 ◻

(ii) between the age of 55 and 65 ◻

(iii) under age 55 ◻

*To some extent heart disease runs in families because of shared inheritance and shared habits*.

**3 Smoking**

a) Never smoked ◻

b) Ex-smoker (gave up > 5 years ago) ◻

c) **Ex-smoker** (gave up less than 5 years ago) ◻

d) Currently **smoke** ◻

*Smoking causes heart disease by increasing the rate at which arteries clog up.To reduce your risk of heart disease it is recommended that you do not smoke at all*.

**4 Blood Pressure**

Are you on medication for high **Yes** ◻ No ◻

blood pressure?

Your blood pressure in mmHg is:

***Referral***

*In normal circumstances The National Heart Foundation recommends BP less than or equal to 135/85. BP may be lowered by making healthy lifestyle changes including losing weight (if necessary), enjoying moderate exercise, not smoking and reducing your intake of fat, alcohol and salt*.

**5 Cholesterol Level**

Fasted ◻ Non Fasted ◻

Are you on medication for high cholesterol levels? **Yes** ◻ **No** ◻

TC cholesterol level is: ▭

HDL ▭

LDL ▭ Ratio ▭ Triglycerides ▭

***Referral***

Causes, usually saturated fats in diet, cholesterol in foods and hereditary factors – FAMILY HISTORY

**LEVELS** Total Cholesterol – 5.5 mmol/L or less

HDL – 1.0 or above LDL – 3.5 or less

Triglycerides – 2.0 or less

Ratio – 4.5 or less over 6.5 BAD

**6 Diabetes**

Do you have Diabetes? **Yes** ◻ **No** ◻

Do you have a family history of Diabetes? **Yes** ◻ **No** s◻

Your blood sugar level is: ▭

Fasted ◻ Non Fasted ◻

***Referral***

Diabetes is a recognized risk factor in developing heart disease. You should have your blood sugar levels checked regularly over the age of 40.Normal levels should be under**6.0 mmol/L (fasting blood sugar).**

**7 Weight**

Self-assessment of your weight range

a) Healthy range ◻

**b) Overweight** ◻

**c) Obese** ◻

*Reducing weight decreases the risk of heart disease by lowering blood cholesterol levels. Being overweight, however, is a risk factor independent of blood pressure and cholesterol*.

Height______________ Weight______________ Waist______________ (Under 100/94cms Men)

**8 Alcohol**

*Moderate alcohol consumption is 2 standard drinks per day for women, and 4 standard drinks per day for men*.

How would you describe your alcohol consumption?

a) Low ◻

b) Medium ◻

**c) High** ◻

*If you have heart disease you can slow the progress, or reverse it, by adopting a healthy lifestyle and reducing your risk factors*.

**9 Exercise**

a) High intensity exercise – more than 3 hours per week (sport, gym, riding, running) ◻

b) Moderate activity – at least 3 hours per week (walking, gardening, work about the house & using stairs) ◻

c) **Moderate activity** less than 3 hours per week ◻

d) **Inactive** throughout the whole week ◻

*Note, during high intensity exercise you should be puffing and notice your heart rate has increased*.

*Any regular exercise helps to protect against coronary heart disease by improving fitness, helping to maintain weight and reducing stress*.

**10 Stress**

*There are many physical, psychological and behavioural symptoms you can experience if faced with high levels of stress*.

How do you describe your stress level?

**a)** Low (Generally happy, I don’t get stressed) ◻

b) Moderate (Sometimes concerned about work and family matters, I have strategies to relax such as exercise) ◻

**c) High** (Under pressure most of the time, I tend to drink alcohol, smoke or not sleep when I feel stressed) ◻

*Exercises or meditation are two ways you can help reduce your stress levels. Walking is recommended for all ages*.

**Your Health Record is your guide of the results of your screening. Please keep this in a safe place or in your wallet.**

**Risk Factors – this is a guide only, to assist you to understand the risk factors associated with heart disease and how best you need to change your lifestyle to reduce your risk.**

**You can reduce your risk of a heart attack with a healthy diet, enjoying regular exercise, not smoking and regular health checks**.

*This form is based on the work done by the National Heart Foundation of Australia in producing the original brochure called Heart Health*.

**Table:** RISK FACTORS (Guide Only)

Age and Sex	
Family History	
**Smoking**	
**Blood Pressure**	
**Cholesterol**	
**Diabetes**	
**Weight**	
**Exercise**	
**Stress**	
**Alcohol**	

**Federally Funded by Department of Health & Ageing**

**In partnership with Hepburn Health Service**

**and Point of Care Unit Flinders University 2006**

**Form no: CIL.PRO.7.2/7/2.Fiia&b**

**Version: 2**

**Developed/Authorized by: Bernard Denner**

 © A CAMH Resource, Mildura, Victoria, 2006

***Early Intervention Screening Program***

**Date:**

    ______________________

**Session at:**

      ______________________

**Date of Birth:**

       ______________________

Male ◻ Female ◻

I consent to my Screening Results being

tracked for the Rural Men’s Health Project Evaluation.

Signed: _____________

Date: _________

**Rural Men’s Health Project** ***Resource developed by CAMH*** ***Based on CAMH Projects:*** ***Diabetes Management across the Mallee Track, Ouyen*** ***& Heart Disease Project, Daylesford*** **Funded by the Department of Health & Ageing Email: bernard@mannet.com.au**
